# Unraveling the Mysteries of Hypoglycemia: A Case Report on Recurrent Hypoglycemic Episodes

**DOI:** 10.1093/jalm/jfaf169

**Published:** 2025-12-02

**Authors:** Tatiana C Coverdell, Caroline E Nottingham

**Affiliations:** Department of Laboratory Medicine, Clinical Center, National Institutes of Health, Bethesda, MD, United States; Department of Laboratory Medicine, Clinical Center, National Institutes of Health, Bethesda, MD, United States

## CASE DESCRIPTION

A 16-year-old female with congenital generalized lipodystrophy (CGL), complicated by hypertriglyceridemia, lipoatrophic diabetes mellitus, and steatohepatitis, presented with recent recurring episodes of overnight hypoglycemia. The patient was prescribed U500 insulin (300 units twice daily, prebreakfast and predinner), canagliflozin (100 mg daily), and metformin (1000 mg twice daily) for glucose management, and reported adherence. A history of poor glucose control was noted, but she had no prior history of hypoglycemic episodes; her most recent hemoglobin A_1c_ was 12.8% (116 mmol/mol) [RI, 4.0%–6.0% (20–42 mmol/mol)] approximately 1 month prior. To determine whether insulin dose adjustments were necessary, she was instructed to complete a glucose diary and log daily glucose values at home using her fingerstick glucose meter.

The glucose diary was completed for 6 weeks and results reported to the clinical team (Table 1). During week 1, recurring morning hypoglycemia was treated by decreasing the patient’s predinner insulin dose, progressing to complete discontinuation of predinner insulin by week 2 (see footnotes in Table 1 corresponding to dose adjustments). Continued morning hypoglycemia in week 4 resulted in a similar dose adjustment for the patient’s prebreakfast insulin dose, leading to discontinuation of all insulin administration by week 5. However, the patient continued to report worsening and more frequent hypoglycemia to a level of severity that required emergency medical services to be contacted. The patient was instructed to discontinue canagliflozin and metformin immediately.

**Table 1.** Patient’s glucose diary.

Week/Day	Blood glucose result (mg/dL^a^)	
	Prebreakfast	Predinner
Week 1		
Day 1	70	101
Day 2	72	168^b^
Day 3	121	—
Day 4	78	167^c^
Day 5	64	163^d^
Day 6	72	−^e^
Week 2		
Day 1	62	approximately 100–160
Day 2	71	approximately 100–160^f^
Day 3	36	−^g^
Day 4	77	—
Day 5	123	—
Day 6	137	—
Week 3		
Day 1	190	112
Day 2	130	—
Day 3	70	—
Day 4	102	92
Day 5	70	—
Day 6	111	—
Week 4		
Day 1	78^h^	—
Day 2	111	—
Day 3	92	—
Day 4	115	121
Day 5	54	66
Day 6	67^i^	90
Week 5		
Day 1	62^j^	81
Day 2	105	—
Day 3	84^k^	80
Day 4	67	84^l^
Week 6		
Day 1	67	—
Day 2	89	118
Day 3	85	—
Day 4	75	—
Day 5	79	66
Day 6	72	103
Day 7	< 40^m^	63

^a^Conversion to SI units: mg/dL × 0.0555 = mmol/L.

^b^Predinner insulin dose changed from 300 units to 250 units.

^c^Predinner insulin dose changed from 250 units to 200 units.

^d^Predinner insulin dose changed from 200 units to 150 units.

^e^Predinner insulin dose changed from 150 units to 100 units.

^f^Predinner insulin dose changed from 100 units to 50 units.

^g^Predinner insulin dose discontinued.

^h^Prebreakfast insulin dose changed from 300 units to 225 units.

^i^Prebreakfast insulin dose changed from 225 units to 200 units.

^j^Prebreakfast insulin dose changed from 200 units to 150 units.

^k^Prebreakfast insulin dose changed from 150 units to 100 units.

^l^Prebreakfast insulin dose discontinued.

^m^Emergency medical services called to patient’s home; canagliflozin and metformin discontinued.

To diagnose the underlying cause of her hypoglycemia, the patient was admitted for an inpatient overnight fast and adrenocorticotrophic hormone (ACTH) stimulation test. During the overnight fast, at 2:45 AM the patient’s continuous glucose monitor alarmed for hypoglycemia and the patient noted shakiness with tremor. Point-of-care glucose was 40 mg/dL (2.2 mmol/L) and the clinical team decided to discontinue the fast. The patient was provided food and drink, and her glucose returned to 70 mg/dL (3.9 mmol/L). Testing resumed with an ACTH stimulation test administered at 8:00 AM. A summary of laboratory values collected during the studies is shown in Table 2 (Overnight fast #1, top).

**Table 2.** Laboratory results from inpatient studies.

		Glucose, mg/dL^a^		Insulin^d^, µU/mL^a^	C-Peptide^d^, ng/mL^a^	Proinsulin^e^, pmol/L	Cortisol^c^, µg/dL^a^	ACTH^f^, pg/mL^a^	Hypoglycemic agent screen^g^
Date/Time		POCT^b^	Lab^c^						
**Reference interval**		74–99	70–99	2.4–24.9	1.1–5.0	3.6–22	<10 AM: 3.7–19.4, >5 PM: 2.9–17.3	5.0–46.0	Negative
**Overnight fast #1**									
Day 1									
11:00 PM		102	49	816	<0.1	1.9	5.4	—	Negative
Day 2									
12:45 AM		59	45	Hemolyzed	<0.1	—	—	—	—
1:45 AM		72	43	>1000	<0.1	—	—	—	—
2:45 AM		40	38	—	—	—	—	—	—
3:20 AM		70	—	Hemolyzed	<0.1	<0.6	11.0	—	Negative
8:00 AM		90	—	—	—	—	—	—	—
**ACTH stimulation test**									
Day 2									
8:32 AM							8.7	30.2	
9:02 AM							16.5	—	
9:32 AM							15.2	—	
**Overnight fast #2**									
Day 2									
6:00 PM		323	325	101.8	1.7				
10:00 PM		352	366	66.2	3.3				
Day 3									
2:15 AM		—	214	Hemolyzed	1.4				
6:00 AM		—	218	27.3	2.5				
9:30 AM		172	175	26.2	2.7				

^a^Conversion to SI units: glucose, mg/dL × 0.0555 = mmol/L; insulin, µU/mL × 6 = pmol/L; C-peptide, ng/mL × 0.33 = nmol/L; cortisol, µg/dL × 27.6 = nmol/L; ACTH, pg/mL × 0.22 = pmol/L.

^b^Nova StatStrip.

^c^Abbott Architect.

^d^Roche Cobas.

^e^MesoScale Discovery.

^f^Siemens Immulite.

^g^LC-MS/MS.

Of note, results for 2 of the 4 serial insulin measurements (12:54 AM and 3:19 AM) were not reported due to hemolysis. The clinical team contacted the laboratory to discuss further.

QUESTIONS TO CONSIDER

1. What is congenital generalized lipodystrophy?
2. What may be the cause of the patient’s recurrent hypoglycemia?
3. What should be considered in laboratory evaluation of hypoglycemia?
4. Why is hemolysis a concern in laboratory testing?

## CASE DISCUSSION

### Congenital Generalized Lipodystrophy

CGL is a rare autosomal recessive disorder (affecting <1 per 1 000 000) associated with 4 distinct mutations: *CGL1* [1-acylglycerol-3-phosphate *O*-acyltransferase 2 (AGPAT2) gene mutation], *CGL2* [BSCL2 lipid droplet biogenesis associated, seipin (BSCL2) gene mutation], *CGL3* [caveolin 1 (CAV1) gene mutation], and *CGL4* [caveolae associated protein 1 (CAVIN1) gene mutation], all of which are characterized by an almost complete absence of subcutaneous adipose tissue and severe deficiency of the adipocyte-derived hormone leptin (1). In CGL, lipid storage is rerouted to ectopic locations, such as the liver and muscles, which causes metabolic abnormalities including insulin resistance, hyperlipidemia, and cirrhosis (1, 2). The patient described in this case had *CGL2*, which is considered the most severe mutation due to lack of mechanical adipose tissue and bone marrow fat, and is associated with an earlier onset of diabetes mellitus (1). The patient presented with multiple metabolic complications arising from *CGL2*, hypertriglyceridemia, diabetes, and metabolic dysfunction-associated steatotic liver disease, and was previously prescribed insulin, canagliflozin, and metformin to manage glycemia.

### Causes of Hypoglycemia

Hypoglycemia is most commonly seen in diabetic patients undergoing insulin therapy or pharmacological interventions that stimulate insulin release from the pancreas (3). Other medications, such as metformin, glucagon-like peptide-1 receptor agonists, sodium-glucose co-transporter 2 inhibitors, and dipeptidyl peptidase-4 inhibitors, can also cause hypoglycemia, but this is less common (3). Although rare in nondiabetic individuals with normal hepatic function, hypoglycemia can be triggered by other medical conditions, but iatrogenic causes such as surreptitious medication should also be considered. Critical illness results in glucose utilization that exceeds intake and production, and is more commonly seen in cases such as sepsis, starvation, renal failure, and end stage liver disease. Excessive alcohol consumption can lead to hypoglycemia primarily by inhibiting gluconeogenesis, although this risk is greater for patients with diabetes on glucose-lowering medications. Nonislet cell tumors cause metabolic derangement through excess secretion of substances that interfere with normal glucose regulation, such as insulin-like growth factor 2, which leads to an increased glucose uptake and sustained hypoglycemia, and islet cell tumors secrete insulin, often in conjunction with inhibition of gluconeogenesis and glycogenolysis, with presentation as morning hypoglycemia (3). Given the patient’s persistent morning hypoglycemia despite discontinuation of insulin and glucose-lowering drugs, additional laboratory testing was ordered to further understand the etiology.

### Laboratory Evaluation of Hypoglycemia

In the laboratory setting, hypoglycemia is often defined as blood glucose <70 mg/dL (<3.9 mmol/L) and can be categorized into 3 levels of severity: Level 1, glucose between 54–70 mg/dL (3.0–3.9 mmol/L); Level 2, <54 mg/dL (<3.0 mmol/L); and Level 3, which includes severe events that requires treatment (4). In cases of hypoglycemia related to diabetic therapy, glucose self-monitoring and ingestion of carbohydrates, including glucagon or glucose, should be used to manage acute hypoglycemic episodes.

Insulin is produced by pancreatic beta cells, initially as preproinsulin that is then cleaved to produce a single insulin peptide and C-peptide (4). Thus, evaluation of insulin, glucose, and C-peptide can be used to differentiate between the presence of endogenous and exogenous insulin, as exogenous insulin does not contain C-peptide (5, 6). In tandem, oral hypoglycemic agent screens can be used to evaluate presence of glucose-lowering medications, typically targeting detection of sulfonylureas and meglitinides.

The workup for individuals without diabetes is more extensive. Insulin suppresses the production of ketones, so laboratory evaluation of ketone bodies, specifically beta-hydroxybutyrate, suggests noninsulin mediated hypoglycemia as seen in starvation (5). Islet cell tumors release increased amounts of proinsulin and exhibit decreased conversion of proinsulin to insulin; detection of higher proportions of proinsulin compared to insulin and C-peptide, along with abnormally increased insulin and C-peptide, can suggest the presence of insulinoma but further imaging studies are required for definitive diagnosis (5).

Evaluation of cortisol, ACTH, and growth hormone, all of which are key regulators of blood glucose, should be used to investigate potential hormone deficiency. An ACTH stimulation test can be performed to assess adrenal function, during which synthetic ACTH (cosyntropin) is administered and cortisol is measured at baseline, 30, and 60 minutes (7). In the context of blood glucose control, cortisol stimulates gluconeogenesis; thus, adrenal insufficiency can present as hypoglycemia, particularly in children (7). An appropriate rise of cortisol on stimulation indicates that adrenal insufficiency is unlikely.

In this case, the clinical team ordered serial glucose, insulin, and C-peptide measurement throughout the patient’s overnight fast, an ACTH stimulation test, as well as proinsulin, cortisol, and a hypoglycemic agent screen (Table 2).

### Hemolysis Interference

Hemolysis is defined as the lysis of red blood cells and subsequent release of their contents into surrounding fluid and is well-defined as a significant interference in immunoassays (8, 9). Hemolysis can interfere through many mechanisms, including spectrophotometric interference with wavelength absorbances similar to hemoglobin, sample dilution effects, and/or release of intracellular contents into the sample (10).

In this case, the laboratory was contacted to release insulin results that were marked as hemolyzed in the patient’s chart. Insulin is particularly sensitive to hemolysis due to release of insulin-degrading peptidases released from erythrocytes that cause a time-dependent decrease in insulin concentrations (11). The degree of insulin reduction is positively correlated with temperature, time, and amount of hemolysis (9).

## CASE RESOLUTION

The laboratory director reviewed results for hemolyzed, unreported specimens. Insulin was analyzed using a Roche cobas 6000 analyzer (e 601 module) which has a hemolysis (H-) index cutoff of 100. Both hemolyzed specimens had H-indices >100 (12:45 AM, H-index 165; 3:20 AM, H-index 475). The director confirmed that insulin results be withheld due to the potential for falsely decreased insulin, which could confound the patient’s evaluation (e.g., endogenous vs exogenous hypoglycemia) and lead to misdiagnosis and unnecessary additional testing.

Using available laboratory results from the overnight fast, the clinical team presumed exogenous insulin was administered. C-peptide remained low [<0.1 ng/mL (<0.033 nmol/L); RI, 1.1–5.0 ng/mL (0.363–1.65 nmol/L)] despite elevated insulin [816 to >1000 µU/mL (4896 to >6000 pmol/L); RI, 2.4–24.9 µU/mL (14.4–149.4 pmol/L)] in nonhemolyzed specimens throughout the duration of the fast. When questioned, the patient denied possessing or using insulin. However, the patient’s father shared that he had seen an insulin vial and syringe among the patient’s belongings, which was confirmed on inspection. A psychiatry consult was requested, and a second overnight fast was scheduled with full-time observation of the patient.

Results from the second overnight fast demonstrated a rise in glucose to >300 mg/dL (>16.7 mmol/L) for >4 hours, for which canagliflozin and metformin were administered. The continuous glucose monitor alerted for persistent hyperglycemia and the patient was given a single dose of insulin. Repeat glucose values decreased to approximately 200 mg/dL (11.1 mmol/L) by 2:00 AM. A one-to-one observer remained in the patient’s room throughout the fast and for the remainder of her hospitalization. Results suggested surreptitious administration of exogenous insulin leading to recurrent, severe hypoglycemic episodes with a subsequent diagnosis of iatrogenic hypoglycemia. With patient and parental agreement, it was decided that all patient medications would be locked up to be accessed and administered only by parents. Insulin, canagliflozin, and metformin were resumed, and the patient was monitored for the remainder of her hospitalization (5 days) during which no additional hypoglycemic events (<70 mg/dL [<3.9 mmol/L]) were noted. In the following year, no further hypoglycemic events were reported.

EDUCATIONAL POINTS

- CGL is a rare genetic disorder characterized by an almost complete lack of body fat with complications such as insulin resistance, diabetes, hypertriglyceridemia, and nonalcoholic fatty liver disease.
- Glucose, insulin, and C-peptide testing can be utilized to assess causes of acute hypo- or hyperglycemic episodes; hemoglobin A_1c_ testing provides valuable information regarding diabetes management over time.
- Insulin assays are sensitive to hemolysis due to degradation of insulin via peptidases released from red blood cells.

## Notes

**Human Genes:**  *AGPAT2*, 1-acylglycerol-3-phosphate O-acyltransferase 2; *BSCL2*, lipid droplet biogenesis associated, seipin; *CAV1*, caveolin 1; *CAVIN1*, caveolae associated protein 1.

**Author Contributions:**  *The corresponding author takes full responsibility that all authors on this publication have met the following required criteria of eligibility for authorship: (a) significant contributions to the conception and design, acquisition of data, or analysis and interpretation of data; (b) drafting or revising the article for intellectual content; (c) final approval of the published article; and (d) agreement to be accountable for all aspects of the article thus ensuring that questions related to the accuracy or integrity of any part of the article are appropriately investigated and resolved. Nobody who qualifies for authorship has been omitted from the list.*

**Authors’ Disclosures or Potential Conflicts of Interest:**  *Upon manuscript submission, all authors completed the author disclosure form.*

**Research Funding:** This research was supported by the Intramural Research Program of the National Institutes of Health (NIH).

**Disclosures:** None declared.

## References

[1] Lightbourne M, Brown RJ. Genetics of lipodystrophy. Endocrinol Metab Clin North Am 2017;46:539–54. 28476236 10.1016/j.ecl.2017.01.012 PMC5424609

[2] Brown RJ, Gorden P. Leptin therapy in patients with lipodystrophy and syndromic insulin resistance. In: Dagogo-Jack S, editors. Leptin regulation and clinical applications. Memphis (TN): Springer; 2015. p. 225–36.

[3] Mathew P, Thoppil D. Hypoglycemia. In: Statpearls [internet]. Treasure Island (FL): StatPearls Publishing; 2024.

[4] Steiner DF, Cunningham D, Spigelman L, Aten B. Insulin biosynthesis: evidence for a precursor. Science 1967;157:697–700. 4291105 10.1126/science.157.3789.697

[5] Sacks DB, Arnold M, Bakris GL, Bruns DE, Horvath AR, Lernmark A, et al Guidelines and recommendations for laboratory analysis in the diagnosis and management of diabetes mellitus. Clin Chem 2023;69:808–68. 37473453 10.1093/clinchem/hvad080 PMC12376302

[6] Leighton E, Sainsbury CA, Jones GC. A practical review of c-peptide testing in diabetes. Diabetes Ther 2017;8:475–87. 28484968 10.1007/s13300-017-0265-4 PMC5446389

[7] Bertholf RL, Cooper M, Winter WE. Adrenal cortex. In: Rifai N, Chiu RWK, Young I, Burnham CD, Wittwer CT, editors. Tietz textbook of laboratory medicine. 7th Ed. St. Louis (MO): Elsevier; 2023. p. 2726–52.

[8] Karin A, Higgins V, Miller J, Brinc D, Kulasingam V, Selvaratnam R. Evaluation of hemolysis, lipemia, and icterus interference with common clinical immunoassays. Clin Chem Lab Med 2023;61:1035–45. 36635939 10.1515/cclm-2022-0924

[9] Garinet S, Fellahi S, Marlin G, Capeau J, Lefèvre G, Bastard JP. Differential interferences of hemoglobin and hemolysis on insulin assay with the Abbott architect-Ci8200 immunoassay. Clin Biochem 2014;47:445–7. 24486652 10.1016/j.clinbiochem.2014.01.026

[10] Simundic A, Dukić L, Biljak VR. Preanalytical variation and pre-examination process. In: Rifai N, Chiu RWK, Young I, Burnham CD, editors. Tietz textbook of laboratory medicine. 7th Ed. St. Louis (MO): Elsevier; 2023. p. 80–128.

[11] Chevenne D, Letailleur A, Trivin F, Porquet D. Effect of hemolysis on the concentration of insulin in serum determined by RIA and IRMA. Clin Chem 1998;44:354–6. 9474041

